# Magnitude of cesarean-section and associated factors among diabetic mothers in Tikur Anbessa Specialized Hospital, Addis Ababa, Ethiopia: A cross-sectional study

**DOI:** 10.3389/fpubh.2022.888935

**Published:** 2022-09-16

**Authors:** Bajrond Eshetu, Bikila Balis, Woreknesh Daba, Bazie Mekonnen, Tamirat Getachew, Ephrem Yohanes Roga, Sisay Habte, Habtamu Bekele, Indeshaw Ketema, Adera Debella

**Affiliations:** ^1^Department of Midwifery, School of Nursing and Midwifery, College of Health and Medical Sciences, Haramaya University, Harar, Ethiopia; ^2^Department of Nursing, School of Nursing and Midwifery, College of Health and Medical Sciences, Addis Ababa University, Addis Ababa, Ethiopia; ^3^Department of Midwifery, School of Nursing and Midwifery, College of Health and Medical Sciences, Ambo University, Ambo, Ethiopia; ^4^Department of Nursing, School of Nursing and Midwifery, College of Health and Medical Sciences, Haramaya University, Harar, Ethiopia; ^5^Department of Emergency and Critical Care Nursing, School of Nursing and Midwifery, College of Health and Medical Sciences, Haramaya University, Harar, Ethiopia

**Keywords:** diabetes mellitus, cesarean-section, mothers, Tikur Anbessa, Ethiopia

## Abstract

**Background:**

Gestational diabetes is associated with multiple adverse pregnancy outcome as a result of unfavorable labor and delivery process with a consequent increase in obstetric interventions including cesarean-section. Even though diabetes mellitus increases the cesarean-section rate; there is no study conducted in Ethiopia. therefore, this study aimed to assess the magnitude of cesarean-section and associated factors among diabetic mothers in Tikur Ambessa Specialize Hospital, Addis Ababa, Ethiopia.

**Methods:**

A facility-based retrospective cross-sectional study was conducted in Tikur Anbessa Specialized Hospital from 1 February to 30 April 2018 among 346 diabetic mothers. All required data were extracted from patients' charts using checklists, and incomplete records were excluded. The collected data were entered into Epi data version 4.2 and exported to SPSS version 20 for analysis. Multiple logistic regression models were fitted to identify factors associated with cesarean section. Adjusted odds ratios along with 95% CI were estimated to measure the strength of the association and declared statistical significance at a *p*-value <0.05.

**Results:**

The magnitude of cesarean-section was 57.8% (95% CI: 51.7, 63.3). Pregnancy-induced hypertension [AOR: 3.35, (95% CI: (1.22, 9.20)], previous C/S [AOR: 1.62, (95% CI: (2.54, 4.83)], and fetal distress [AOR: 4.36, (95% CI: 1.30, 14.62)] were factors significantly associated with cesarean-section.

**Conclusion:**

A considerable number of diabetic mothers gave birth by cesarean-section. Pregnancy-induced hypertension, previous cesarean-section, and fetal distress were factors more likely to increase the rate of cesarean-section. Most of the factors were modifiable by following the WHO recommendation for cesarean-section.

## Introduction

Worldwide, more than 422 million people are affected by diabetes mellitus (DM), and more in low- and middle-income countries (1). According to the International Diabetes Federation (IDF), gestational diabetes is associated with multiple adverse pregnancy outcomes where 20.9 million or 16% of live births had some form of hyperglycemia (2).

In addition, DM poses multiple risks to pregnant women and their offspring, such as preeclampsia, macrosomia, prematurity, obstructed labor, shoulder dystocia, congenital anomalies, birth injuries, and a consequent increase in obstetric interventions including cesarean-section (3, 4). Cesarean-section (CS) is a surgical procedure indicated when vaginal delivery presents a higher likelihood of adverse maternal and/or perinatal outcomes (5, 6).

Worldwide, one in five diabetic mothers give childbirths by CS (7). In Ethiopia, the rate of CS among general populations varies between regions with a range of 0.4% in Somali to 21.4% in Addis Ababa (8), with a national pooled prevalence of 29.55% (9). However, WHO recommendation for CS is 5–15% to have an optimal impact (6). Moreover, although CS is taken to relieve obstructed labor, it has complications of wound dehiscence and ulcer in addition to other complications that occur during the operative and postoperative period (10, 11).

Various characteristic features have been identified as risk factors for CS in women with DM, namely, maternal age, marital status, parity, history of stillbirth, CS scar, macrosomic infant, obstructed labor, and fetal distress (5, 12–16). Identifying the magnitude of CS and related factors among diabetic mothers is vital to generating new evidence, and developing contextual interventions. Nevertheless, there is no information regarding CS among diabetic mothers in Ethiopia. Therefore, this study aimed to assess the magnitude of CS and its associated factors among diabetes mothers in Addis Ababa, Ethiopia

## Methods and materials

### Study setting, design, and period

A facility-based retrospective cross-sectional study was conducted from 1 February to 30 April 2018, in the Tikur Anbessa Specialized Hospital found in Addis Ababa the capital city of Ethiopia. Tikur Anbessa is Ethiopia's largest specialized and referral public hospital. According to the 2007 statistical report of the population and housing census of Ethiopia, Addis Ababa has a total population of 3,384,569 (17). The health service coverage of the city is 52.2, and 82% of deliveries take place in public health facilities (18). There are 17 public and 25 private hospitals (19). The hospital provides diagnoses and treatment for approximately 370,000–400,000 patients per year in all wards. The diabetes center is one of the departments in the hospital services. Of a total of 800 beds, 80 were used by the department of obstetrics and gynecology during the survey (20). In the city, around 4,600 deliveries were attended each year of which 60% are operative deliveries (8).

### Sample size determination

Sample size was determined using a single population proportion formula. The following assumptions were used while calculating the sample size; 95% confidence level, a margin of error (0.05) and 50% anticipated population proportion was taken since there is no published paper on assessing cesarean-section among mothers with diabetes in Ethiopia. Therefore, 422 sample size was planned to use for this study with a 10% non-respondent rate. However, since the numbers of diabetic mothers' cards with complete records were less than the planned sample size, all cards with complete records were included.

### Sampling technique and study population

Tikur Anbessa Specialized Hospital was selected purposively since it is the largest public hospital with maternal health service and diabetes center/department. The required data were extracted from patient charts. First, the health management and information system (HMIS) delivery registration book, postnatal registrations, admission registration, and gestational diabetes registration in diabetes mellitus center card numbers were obtained and documents of all delivered mothers who had DM during the planned study period at obstetrics ward was searched and checked for completeness of the data. Then, cards of mothers with complete records were separated and counted. Since the numbers of cards with complete records were less than the planned sample size, all cards of the diabetic mothers with completed records were included whereas incomplete documents were excluded.

### Data collection tools and procedures

A structured checklist adapted from published studies with certain modifications was used (16, 21–23). The checklist includes information on socio-demographic characteristics, obstetric characteristics, feto-maternal outcome, and diabetes mellitus. After the card numbers were obtained, the documents of all delivered mothers who had DM during the planned study period at the obstetrics and gynecologic ward were searched and checked for completeness of the data. The data were collected through document review in the obstetrics ward from the patient chart, delivery registration book, post-natal registrations, duty report registration books, and operation logbooks. The data were extracted by two record office staff, three midwives working in the obstetrics unit, two supervisors, and the principal investigator.

### Data quality control

To ensure the quality of the data before the actual data collection, a pretest was done on 5% of patient records at Zewditu Memorial Hospital. Appropriate modifications were made to the checklist and procedures after analyzing the pretest result. One-day training was given to data collectors on how to collect the data. The supervisors and the principal investigator coordinated and checked the data collection process, and daily supervision was done to ensure the completeness and consistency of the gathered information.

### Data processing and analysis

The collected data was entered to Epi data version 4.2, cleaned, and transported to SPSS version 23.0 for data analysis. Descriptive statistics, frequency tables, figures, and percentages were used to summarize the data. Bi-variable analysis and multiple logistic regressions were used to test for the association between dependent and independent variables. Variables that showed an association in the bi-variable analysis with *p*-value < 0.25 were entered into a multiple logistic regression model. At last, the multiple logistic regression models were used after controlling for confounding factors using regression. Hosmer-Lemeshow's goodness of fit test was used to assess whether the necessary assumptions for the application of multiple logistic regression had been fulfilled. Multi-co-linearity was assessed by using standard error, and the variables were entered into the multiple models without multi-co-linearity. Adjusted odds ratios (AOR) were calculated with 95% CIs, and statistical significance was declared at *p*-value < 0.05.

### Ethical considerations

Ethical clearance was obtained from the ethical review committee of Addis Ababa University, School of Nursing and Midwifery. The official letter of cooperation was taken from Addis Ababa University to Tikur Anbessa Specialized Hospital. Informed and written consent was obtained from the medical director and NICU head. To keep confidentiality, their patients were not documented; rather a code was given for each card.

## Results

### Socio-demographic characteristics of the mothers

In this study, a total of 346 records were reviewed. Of a total of the respondents, the majority (40.2%) were in the 30–34 years age group. Almost all (98.6%) mothers were married. Addis Ababa was the dominant residence of the mothers, accounting for 93.1%. Regarding the occupational status of the mothers, almost half of them (50.2%) were housewives (Table 1).

**Table 1:** Socio-demographic characteristics of the diabetic mothers delivered at the Tikur Anbessa Specialized Hospital Addis Ababa, Ethiopia, 2018.

**Variables**	**Category**	**Frequency**	**Percent**
		**(*n* = 346)**	**(%)**
Age in years	18–20	8	2.3
	20–24	24	6.9
	25–29	92	26.6
	30–34	139	40.2
	>35	83	24
Marital status	Unmarried	5	1.4
	Married	341	98.6
Occupation	House wife	174	50.2
	Gov't employees	172	49.8
Address	Addis Ababa	322	93.1
	Out of Addis Ababa	24	6.9
BMI	Normal	160	46.2
	Over weight	186	53.8

### Obstetrics characteristics of the mothers

Regarding the parity of diabetic women, the majority (80.9%) were multipara. Nearly one out of five, (20.8%) of mothers had a history of abortion and CS (20.2%), respectively. Around (13.6%) of mothers had experienced a stillbirth in their previous pregnancy (Table 2).

**Table 2:** Obstetrics history of diabetic mothers delivered at the Tikur Anbessa Specialized Hospital in Addis Ababa, Ethiopia, 2018.

**Variables**	**Category**	**Frequency**	**Percent**
		**(*n* = 346)**	**(%)**
Parity	Primipara	66	19.1
	Multipara	280	80.9
History of abortion	Yes	72	20.8
	No	274	79.2
History of CS	Yes	70	20.2
	No	276	79.8
History of stillbirth	Yes	47	13.6
	No	299	86.4
History PIH	Yes	32	9.2
	No	314	89.8
History of birth weight>4 kg	Yes	28	8.1
	No	318	90.9

Nearly half, (51.2%) of the labors occurred spontaneously and (30.3%) were elective CS. Pregnancy-induced hypertension (PIH) accounts for the major parts of the complications the mother developed from pregnancy to postpartum period (26.0%). The majority, (82.1%) of mothers gave birth at term, and 97.4% of delivered newborns were alive (Table 3).

**Table 3:** Current obstetrics characteristics of diabetic mothers delivered at the Tikur Ambessa Specialized Hospital in Addis Ababa, Ethiopia, 2018.

**Variables**	**Category**	**Frequency**	**Percent**
		**(*n* = 346)**	**(%)**
Onset of labor	Spontaneus	177	51.2
	Induced	64	18.5
	Elective CS	105	30.3
GA at time of delivery	Preterm	62	17.9
	Term and above	284	82.1
Maternal Outcome	PIH	90	26.0
	Polyhydroaminus	5	1.4
	Obstructed labor	1	0.3
	Hypotyrodism/cardiac/ renal diseases	10	3.4
	Tear (traumatize labor)	7	2.0
Fetal outcome	Live birth	337	97.4
	Macrosomic baby (≥4 kg)	61	17.6
	Low birth weight (<2.99 kg)	35	10.1
	Respiratory distress	32	9.2
	Hypoglycemia	10	2.9
	IUFD /still birth	9	2.6
	Jaundice	7	2.0
	Birth injury/defect	9	2.6

PIH, Pregnancy Induced Hypertension; IUFD, Intrauterine Fetal Death.

### Mode of delivery

**Figure 1:**
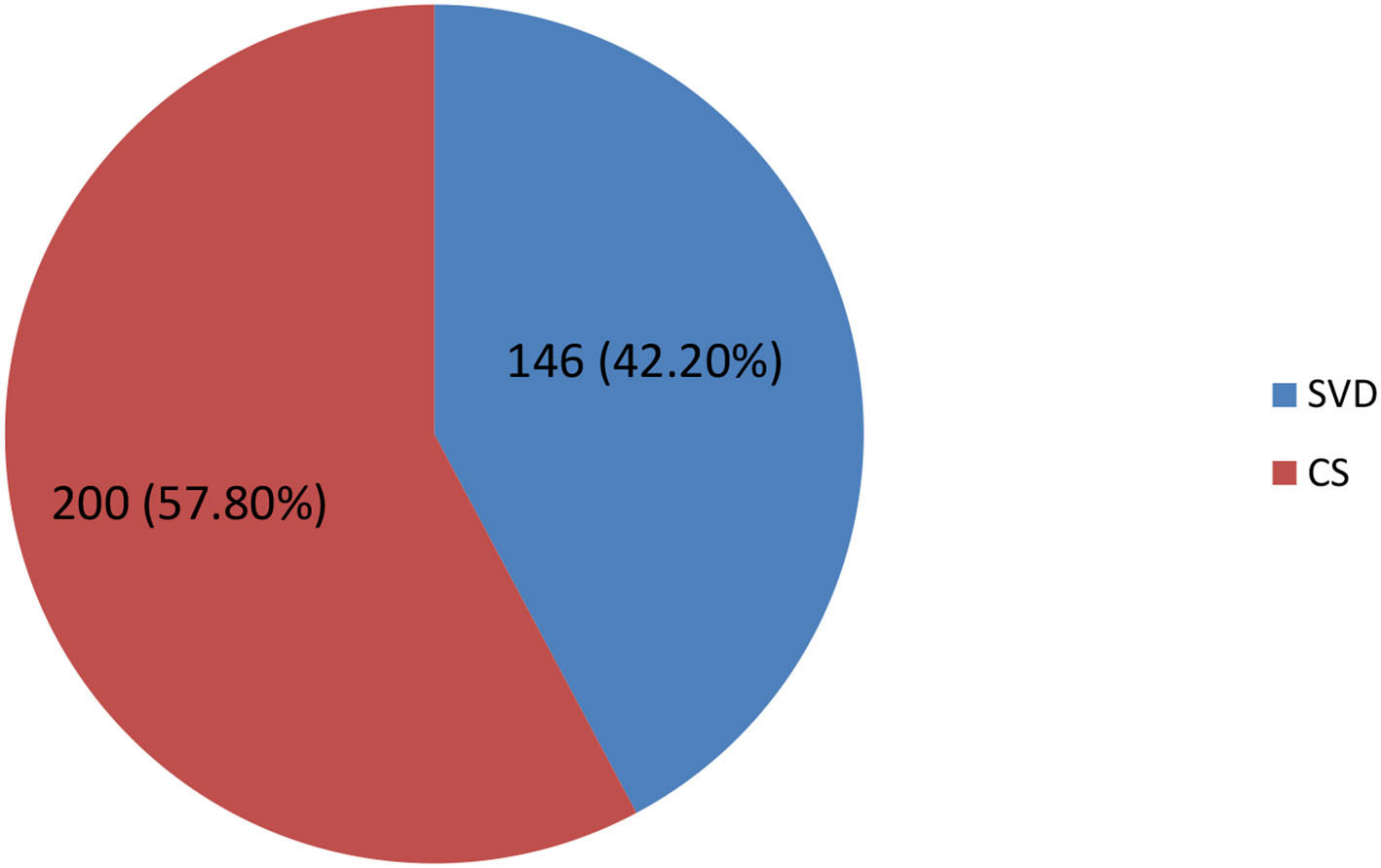
Mode of delivery among diabetic mothers at the Tikur Anbessa Specialized Hospital in Addis Ababa, Ethiopia, 2018.

Regarding mode of delivery, the majority (57.8%) of mothers gave birth by CS whereas the rest (42.2%) of the mothers gave childbirth by spontaneous vaginal delivery (Figure 1).

### Factors associated with cesarean-section

In the bivariate logistic regression analysis, marital status, occupational status, residence, history of stillbirth, parity, history of abortion, PIH, birth weight, and fetal distress were factors significantly associated with CS. However, in multiple logistic regression mothers who have PIH were three times [AOR: 3.35, (95% CI: (1.22, 9.20)] more likely to undergo CS. Also, mothers who have a history of CS were 1.62 times [AOR: 1.62, (95% CI: (2.54, 4.83)] more likely to undergo CS than their counterparts. Mothers who have fetal distress were nearly four times [AOR: 4.36, (95% CI: 1.30, 14.62)] more likely to give birth by CS (Table 4).

**Table 4:** Factors associated with cesarean-section among diabetic mothers delivered at the Tikur Ambessa Specialized Hospital in Addis Ababa, Ethiopia, 2018.

**Variables**	**Category**	**COR [95% CI]**	***P*** **-value**	**AOR [95% CI]**	***P*** **-value**
Marital status	Unmarried	1.00		1.00	1.00
	Married	2.95 (1.32, 26.75)	0.021	0.49 (0.02, 9.53)	0.644
Occupational status	House wife	1.00	1.00	1.00	1.00
	Gov't employee	1.30 (1.05, 2.99)	0.025	1.18 (0.53, 2.60)	0.674
Residence	Addis Ababa (AA)	2.96 (1.07, 8.12)	0.035	0.22 (0.04, 1.15)	0.074
	Outside of AA	1.00	1.00	1.00	1.00
History of stillbirth	Yes	0.53 (0.30,0.92)	0.026	1.30 (0.44, 3.84)	0.634
	No	1.00	1.00	1.00	1.00
Parity	Primipara	1.86 (1.42, 24.16)	0.028	2.92 (0.99, 8.58)	0.052
	Multipara	1.00	1.00	1.00	1.00
History of CS	Yes	0.25 (0.12, 0.46)	0.000	1.62 (2.54, 4.83)	0.001
	No	1.00	1.00	1.00	1.00
PIH	Yes	1.80 (1.38, 3.70)	0.058	3.35 (1.22, 9.20)	0.001
	No	1.00	1.00	1.00	1.00
EFW	<4 kg	1.00	1.00	1.00	1.00
	≥ 4 kg	1.52 (1.22, 8.25)	0.021	0.49 (0.17, 1.40)	0.186
Fetal distress	Yes	2.43 (2.38, 5.47)	0.000	4.36 (1.30, 14.62)	0.007
	No	1.00	1.00	1.00	1.00

## Discussion

In this study, a significant number of diabetic mothers gave birth by CS. Previous CS, PIH, and fetal distress were factors significantly associated with CS.

More than half, 57.8% (95% CI: 51.7, 63.3%) of diabetic mothers gave birth by CS. This is higher than the studies conducted in South Wales (32.0%) (24), Canada (29.1%) (25), Harar (34.3%) (16), Mizan Aman (21.1%) (26), and Dessie (47.6%) (27). The possible reason is the difference in a study population, and the prevalence of primary CS high in these high-risk populations which may increase a rate of repeated CS (28). Diabetes mellitus is associated with obstructed labor and shoulder dystocia which are the most common indication of CS (12, 13, 29).

Mothers who have PIH were 3.35 times more likely to give birth by CS than mothers without PIH. This is supported by a study conducted in Addis Ababa (30) where the odds of CS increase among mothers with PIH. This implies shortening the labor process and delivery per the management protocol of severe preeclampsia and/or eclampsia designated to prevent perinatal morbidity and mortality (6, 31).

In a similar manner, mothers who have previous CS were more likely to give birth by CS than mothers without CS. Similar reports pointed out by Tsega et al. (16) and Gebreegziabher et al. (30) where mothers who gave previous birth by CS were more likely to give current birth by CS. The reason could be the fact that mothers who previously gave birth by CS were more likely to present with permanent indications for CS or a high risk for wound dehiscence (6, 32).

The odds of CS were four times higher among mother with fetal distress than their counterpart. This is supported by a study conducted in Harar (3) where the odds of CS increase among mothers with the fetal distress. In fact, the fetal distress especially that occurs during first stage of labor is one of the absolute indications of CS which is recommended by WHO to save lives of the infants (6).

## Strengths and limitations of the study

The study was used to generate initial data for researchers for further study. However, this study may suffer from incomplete data as it was obtained from secondary data. The other limitation is that the cross-sectional study design cannot establish a temporal relationship between the outcome and response variables.

## Conclusion

This study showed CS among diabetic mothers was high. Mothers with PIH, previous CS, and fetal distress were factors significantly associated with CS. Thus, emphasis should be given to reducing CS among diabetic mothers to reduce the complication of wound dehiscence and ulcer in addition to other complications most probable to occur from CS. Increasing the quality of obstetrics care during pregnancy, intrapartum, and postnatal period among diabetic mothers is crucial to prevent complications of CS. Moreover, improving the utilization of preconception care among diabetic women is crucial in controlling PIH during the perinatal period. Also, CS should be performed based on the indication.

## Data availability statement

The original contributions presented in the study are included in the article/supplementary material, further inquiries can be directed to the corresponding author/s.

## Ethics statement

The studies involving human participants were reviewed and approved by Ethical Review Committee of Addis Ababa University, School of Nursing and Midwifery (aau/chs/mhnsg19/2018). Written and informed consent was taken from head of the inistitutio before data collection.

## Author contributions

BE, WD, and BM: conceptualization, supervision, investigation, methodology, writing—original draft, and writing—review and editing. BB: methodology, data curation, formal analysis, and read and approved the final manuscript. TG, ER, SH, HB, IK, and AD: conceptualization, methodology, writing—original draft, and writing—review and editing. All authors contributed to the article and approved the submitted version.

## Funding

Dire Dawa University has provided financial support for this study. The authors declare that the funding body has no role in designing the study, data collection, data analysis, and writing the manuscript.

## Conflict of interest

The authors declare that the research was conducted in the absence of any commercial or financial relationships that could be construed as a potential conflict of interest.

## Publisher's note

All claims expressed in this article are solely those of the authors and do not necessarily represent those of their affiliated organizations, or those of the publisher, the editors and the reviewers. Any product that may be evaluated in this article, or claim that may be made by its manufacturer, is not guaranteed or endorsed by the publisher.
